# Elamipretide Rejuvenates Oocyte Quality and Restores Female Fertility During Reproductive Aging

**DOI:** 10.1111/acel.70749

**Published:** 2026-10-02

**Authors:** Hao‐Lin Zhang, Yue Wang, Caizhu Wang, Xin Guo, Huanhua Chen, Yu‐Xuan Hou, Xuan Wu, Zi‐Jian Wu, Wen‐Lin Pan, Rui‐Jie Ma, Ping‐Shuang Lu, Jinhui Shu, Shao‐Chen Sun

**Affiliations:** ^1^ College of Animal Science and Technology Nanjing Agricultural University Nanjing China; ^2^ Center of Reproductive Medicine Maternity and Child Health Care of Guangxi Zhuang Autonomous Region Nanning Guangxi China; ^3^ Guangxi Key Laboratory of Artificial Intelligence for Genetic Diseases of Long‐Dwelling Nationalities Affiliated Hospital of Youjiang Medical University for Nationalities Baise Guangxi China

**Keywords:** aging, fertility, oocyte, peptide, reproduction

## Abstract

Aging represents a major contributor to the deterioration of female fertility, with oocyte quality being the central determinant of ovarian aging, a key factor underlying infertility. Although several natural compounds with anti‐aging activity have been documented, highly effective strategies to reverse ovarian aging remain elusive. Here, we report that the peptide elamipretide preserves ovarian function and sustains female fertility during maternal aging. We found that elamipretide administration increases litter size in aged mice and ameliorates oocyte quality decline. Metabolomic and transcriptomic profiling revealed substantial restoration of multiple biological processes in aged oocytes after elamipretide injection. Elamipretide improved both nuclear and cytoplasmic maturation of aged oocytes, accompanied by enhanced cytoskeletal dynamics, mitochondrial metabolism, and organelle reorganization. Mechanistically, elamipretide alleviated age‐associated oocyte maturation defects through synergistic activation of the vitamin B6–VEGF axis. Furthermore, elamipretide significantly promoted maturation, fertilization, and cleavage of aged human oocytes. Elamipretide treatment also rescued the developmental competence of porcine oocytes under oxidative damage. Collectively, this study identifies a novel peptide therapeutic candidate for aging‐related infertility, showing that elamipretide restores the quality of aged oocytes for fertility by coordinately boosting nuclear and cytoplasmic maturation via activation of the VEGF signaling pathway.

## Graphical abstract

We reported a novel peptide therapy showing that elamipretide restores the quality of aged oocytes for fertility by coordinately boosting oocyte nuclear and cytoplasmic maturation via activation of the VEGF signaling pathway.

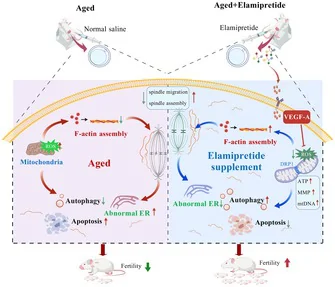

## 1 Introduction

Advanced maternal age (AMA) is associated with a variety of adverse pregnancy outcomes, including miscarriage, preterm birth, fetal growth restriction (FGR), and chromosomal abnormalities (Alamdo et al. 2025; Soares et al. 2025; Swanson and Norton 2025). Women aged 35–39 are more prone to infertility than those in other age groups (Liang et al. 2025; Sauer 2015). Age‐related decline in oocyte quality is a key indicator of ovarian aging and an important cause of reduced fertility in elderly women. Oocyte maturation is a key process that determines the quality of oocytes, and it is complex and precisely regulated (Jiang et al. 2023). The maturation of the oocytes includes two aspects: nuclear maturation and cytoplasmic maturation. The nuclear maturation of oocytes mainly refers to the separation of chromosomes, which reflects the ability of oocytes to resume meiosis; and the cytoplasmic maturation of the oocytes involves the accumulation of mRNAs, proteins, and substrates required for subsequent fertilization/development (Sun and Nagai 2003; Watson 2007). The cytoplasmic maturation failure induced by aging is associated with mitochondrial dysfunction, alterations in mitochondrial biogenesis, changes in mitochondrial morphology, distribution, activity and dynamics, smooth endoplasmic reticulum malformation, calcium disturbance, and cytoskeletal changes (Mao et al. 2014). Previous studies have found oxidative damage in oocytes of AMA and confirmed that these oocytes have mitochondrial dysfunction (Guo et al. 2025). Mitochondria dysfunction is an important reason led to decreased oocyte quality in aged mice or females (Kujoth et al. 2005). Besides, age‐related changes in polar metabolites suggested a decrease in mitochondrial function, as demonstrated by NAD+, purine, and pyrimidine depletion, while glycolysis substrates and glutamine accumulated with age (Smits et al. 2023). Mitochondria are the multifunctional powerhouse of cells, including human oocytes, by producing oxidative phosphorylated ATP (OXPHOS) through five complexes (Complex I–V) in the electron transport chain (ETC, Zong et al. 2016). Thus, mitochondria are key organelles that provide energy for oocyte maturation, fertilization, and embryonic development. In aging ovaries or oocytes, the main feature of mitochondrial dysfunction is the decline in the respiratory capacity of each mitochondrion, while the mitochondrial membrane potential (MMP) drops in a stable state (Miwa et al. 2022). In aging cells, relatively low MMP is typically associated with increased production of reactive oxygen species (ROS), which triggers oxidative stress, in turn leading to nuclear DNA and mitochondrial damage, insufficient intracellular energy supply, calcium homeostasis imbalance, and meiotic spindle abnormalities, ultimately potentially resulting in oocyte aneuploidy (Correia‐Melo et al. 2016; Passos et al. 2007; Rizza et al. 2018).

Multiple studies focused on investigating targeted rescue strategies, including regulating endogenous metabolism, supplementing natural extracts, and applying small‐molecule drug inhibitors for improving the quality of aged oocytes. The small molecule 78c, as an inhibitor of CD38 (ADP‐ribosyl cyclase/hydrolase), was demonstrated to elevate NAD^+^ levels and reduce inflammation in aging ovaries, thereby improving oocyte quality (Yang et al. 2024). While the generation of young follicle‐aged oocyte chimeras improved the developmental quality of embryos following in vitro fertilization and embryo implantation (Wang et al. 2024). Besides, regulation of endogenous metabolism such as mevalonate (MVA) and supplementation of natural extract like 8‐isopentenyl flavone provides (8‐IPF) could promote the synthesis of FPP in adjacent granulosa cells, thereby restoring the assembly of cortical actin in aged oocytes (Liu et al. 2025). In addition, there are relevant studies on using extracts to rescue the quality of aged oocytes. Rapamycin was proven to ameliorate age‐related infertility caused by ribosomal dysfunction via restoring protein homeostasis (Li et al. 2025). It was found that melatonin eliminated oxidative stress‐induced meiotic defects and aneuploidy in aged mouse oocytes (Zhang et al. 2020). Elevating spermidine levels could improve follicular development, oocyte maturation, early embryonic development, as well as female fertility in aged mice (Zhang, Bai, et al. 2023). Additionally, antioxidant interventions specifically directed towards mitochondria represent a key approach in this field. MitoQ and SkQ1, which could effectively reduce ROS generation and preserve mitochondrial structure and function through mitochondria‐targeted antioxidant properties (Andreev‐Andrievskiy et al. 2016; Gonzalo‐Skok and Casuso 2024). Nicotinamide mononucleotide (NMN) and NAD+ enhancers, which could promote cellular metabolism, energy production, and mitochondrial function by increasing the intracellular NAD+ levels (Amjad et al. 2021). CoQ10 mainly functions in the mitochondrial ETC, serving as an electron carrier to transfer electrons from complexes I and II to complex III, driving the proton pump, helping to establish a membrane electrochemical gradient, and promoting ATP synthesis (Silva et al. 2022). However, these therapeutic strategies still suffer from drawbacks such as low targeting efficiency and poor bioavailability. Therefore, it is imperative to explore safer and more readily absorbable small‐molecule agents for clinical translation.

Arg‐Dmt‐Lys‐Phe‐NH2 (also known as MTP‐131, elamipretide, and Bendavia, collectively referred to as elamipretide) is a cell membrane‐penetrating aromatic cationic tetrapeptide with a molecular weight of 639.8 g/mol, composed of four amino acids with both hydrophilic and hydrophobic properties (Daubert et al. 2017). It is reported that elamipretide can improve mitochondrial function and reduce oxidative stress damage (Sabbah et al. 2016). Combining the advantages of elamipretide such as small molecular size, easy synthesis, good water solubility, not easily degraded by peptidase, and high stability in solution, elamipretide is a potential mitochondrial‐targeted drug that can be applied in clinical practice. Under physiological pH conditions, elamipretide carries three positive charges and selectively targets and accumulates in the inner mitochondrial membrane through electrostatic and hydrophobic interactions (Zhu et al. 2022). It has been demonstrated to bind cardiolipin with high affinity. In ischemia reperfusion injury, it formed a complex with cardiolipin on the inner mitochondrial membrane, thereby inhibiting cytochrome *c* peroxidase activity and protecting mitochondrial cristae (Birk et al. 2013). Cardiolipins in mitochondria were prone to oxidation under oxidative stress, resulting in membrane disruption, reduced ETC efficiency, and diminished ATP production (Yin and Zhu 2012). The specific binding of elamipretide to cardiolipin effectively prevented cardiolipin oxidation (Birk et al. 2014). Elamipretide thus stabilized the integrity of mitochondrial membranes and enhanced the efficiency of the ETC, thereby optimizing the overall bioenergetic function of mitochondria (Szeto 2014). Elamipretide could improve mitochondrial structure and function, thereby further reducing the production of reactive oxygen species (ROS) in mitochondria and minimizing the damage of oxidative stress to mitochondria (Mo et al. 2019). Additionally, in human retinal endothelial cells, elamipretide was found that it could also lower abnormal MMP, prevented excessive calcium ions from entering mitochondria, and inhibited cell apoptosis (Li et al. 2011). These effects of elamipretide collectively enhance mitochondrial energy metabolism efficiency, stabilize ATP production, and reduce oxidative stress damage (Chatfield et al. 2019; Cho et al. 2007; Szeto 2008). Report indicated that elamipretide treatment is effective at mitigating signs of sarcopenia and cardiac dysfunction in an aging mouse model (Mitchell et al. 2025). Another study showed long treatment with elamipretide could improve the physical performance of aged males (Nickel et al. 2022).

In this study, we tried to explore whether the treatment of elamipretide could reverse the fertility decline of aging females and the potential mechanism. We found that elamipretide synchronously promotes oocyte nuclear‐cytoplasmic maturation by activating the VB6‐VEGF axis and enhances embryo quality to reverse age‐related fertility decline in mice.

## 2 Materials and Method

### 2.1 Antibodies and Chemicals

Mouse monoclonal anti‐α‐tubulin‐FITC antibody was from Sigma‐Aldrich Corp (St. Louis, MO, USA, Cat# F‐2168‐2ML, 1:400). TRITC‐Phalloidin was purchased from SAITONG (Beijing Pusitang Biotechnology Co. LTD, Cat# T10446‐300 T, 1:200). Rabbit anti‐gamma tubulin antibody, and rabbit anti‐Rab10 antibody were from Abcam (Cambridge, UK, Cat# ab179503, 1:200). Mito‐Tracker Red CMRos (1:500, Cat# M7521, Invitrogen, Eugene, OR, USA) was used to detect the distribution of mitochondria with an ultimate density of 2 μmol/L. TMRE (Cat# C2001S, 1:200) was used to detect the MMP. ROS (Cat# S0033S, 1:800), Annexin‐V (Cat# C1062S, 1:10), ER‐tracker Green (Cat# C1042M‐1, 1:100), and lysosome‐tracker Red (1:12000, Cat# C1046) were purchased from Beyotime Biotechnology (Nantong, China). Rabbit anti‐LC3B antibody was purchased from Cell Signal Technology (Cat #2775, 1:100). GRP78/BIP (11587–1‐AP) was from Proteintech Group Inc. (Rosemont, IL, USA). *Lens Culinaris* Agglutinin (LCA)‐Fluorescein (FITC) was from Invitrogen Corporation (Carlsbad, CA, USA, Cat# L32475). Rabbit monoclonal anti‐VEGF, anti‐PI3K and anti‐AKT2 was purchased from Cell Signal Technology. Beta‐actin and α‐tubulin were purchased from Cell Signal Technology. Goat anti‐rabbit IgG/Alexa Fluor 488 (Cat# ZF‐0511, 1:200) and TRITC‐conjugated goat anti‐rabbit IgG (Cat# ZF‐0316, 1:200) were from Zhongshan Golden Bridge Biotechnology Co. Ltd. (Beijing, China). Horseradish peroxidase‐conjugated goat anti‐rabbit/mouse IgG (H + L) antibodies (Cat# CW0102S, 1:2000) were obtained from CWBIO (Beijing, China). Emvododstat (PTC299) (E1078), LY294002(S1105), and Wortmannin (S2758) was purchased from Selleckchem.com. All other chemicals were purchased from Sigma (St. Louis, MO, USA), unless otherwise stated.

### 2.2 Mouse Oocyte Collection and Culture

The animal experiments in this study were approved by the Animal Ethics Committee of Nanjing Agricultural University and were conducted in strict compliance with the guidelines of the Animal Research Committee. ICR female mice aged 10 months and 6–8 weeks were subjected to superovulation by intraperitoneal injection of 10 IU PMSG, followed by 10 IU hCG 46–48 h later. Cumulus–oocyte complexes were collected from the oviduct ampullae approximately 14 h after hCG administration. Granulosa cells were then removed by hyaluronidase digestion, and the oocytes were washed prior to subsequent experiments. Germinal vesicle (GV)‐stage oocytes were meticulously harvested from the ovaries of both 8, 10, and 12‐month‐old female mice and 6‐ to 8‐week‐old ICR female mice. Following a thorough cleansing procedure, these oocytes were subsequently transferred into M2 culture medium, overlaid with a layer of paraffin oil to maintain a stable microenvironment. The oocytes were then cultured under precisely controlled conditions of 37°C and 5% CO_2_ for durations of 8 and 12 h, respectively. This protocol enabled the successful acquisition of oocytes at the metaphase I (MI) and metaphase II (MII) stages, which were subsequently utilized for further experimental procedures.

### 2.3 In Vitro Fertilization and Embryo Culture

ICR female mice (purchased from Nanjing Medical University) at 10 months and 6–8 weeks of age were induced to superovulate through injection of 10 IU of PMSG, followed by 10 IU of hCG 46–48 h later. Approximately 14 h after hCG injection, cumulus‐oocyte complexes were retrieved from the ampullae of the female mice's oviducts and placed into HTF fertilization drops. ICR male mice (9–10 weeks old) were purchased from Qinglongshan Animal Farm. Their epididymides were harvested and capacitated in prewarmed HIF medium for 1 h at 37°C under 5% CO_2_. Subsequently, capacitated sperm were introduced into the fertilization drops and co‐incubated with oocytes for 4–6 h at 37°C under 5% CO_2_ for fertilization. Finally, the fertilized eggs were collected and cultured in KSOM medium (37°C, 5% CO_2_) until they developed into early embryos, such as the 2‐cell and 4‐cell stages.

### 2.4 Human Oocyte Collection From the Patients

The human oocyte study was approved by the Ethics Committee of Guangxi Maternal and Child Health Hospital and conducted in accordance with the Declaration of Helsinki. Written informed consent was obtained from each participant. All resulting embryos were used solely for research purposes to assess oocyte developmental competence and were neither transferred nor cryopreserved for clinical use. Patients undergoing intracytoplasmic sperm injection (ICSI) cycles were enrolled. The inclusion criteria were: (1) meeting the clinic's indications for ICSI, and (2) at least two germinal vesicle (GV) oocytes retrieved. The exclusion criterion was a maturation rate below 60%.

### 2.5 Randomization and Culture Procedures

Ovarian stimulation was performed using the antagonist protocol. In brief, recombinant FSH was initiated on menstrual cycle Days 2 and 3. A GnRH antagonist (Cetrotide, MSD China) was introduced when follicles reached 11–13 mm and luteinizing hormone (LH) ≥ 10 IU/L or estradiol (E2) ≥ 1570 pmol/L. Human chorionic gonadotropin (hCG, 6000–8000 IU) was administered when at least three follicles reached ≥ 17 mm or at least two follicles reached ≥ 18 mm. Oocytes were collected by transvaginal ultrasound‐guided follicular aspiration 34–36 h after hCG administration. Cumulus‐oocyte complexes (COCs) were promptly transferred to the incubator in commercial cleavage culture medium (Vita, Shenzhen, China). After 3 h of incubation, COCs were denuded using hyaluronidase to remove cumulus cells, and metaphase II (MII) oocytes were selected for ICSI. GV oocytes were collected for in vitro maturation (IVM).

All cultures were performed in a humidified tri‐gas incubator at 37°C with 89% N_2_, 6% CO_2_, and 5% O_2_. GV oocytes were divided into two groups based on patient age: an advanced‐age group (≥ 35 years) and a younger group (< 35 years). Within each age group, GV oocytes were randomly allocated to either the experimental group or the control group. Experimental group: commercial IVM medium supplemented with 500 μM elamipretide; Control group: commercial IVM medium alone. The maturation media were prepared 4–6 h in advance and equilibrated at 37°C in 89% N_2_, 5% O_2_. Both groups were cultured in the same incubator under 37°C in 89% N_2_, 5% O_2_ for observation. Oocytes that extruded the first polar body were defined as mature MII oocytes. Mature oocytes (MII) obtained at 24 h were subjected to ICSI using surplus sperm sample donated for research purposes, and this day was designated as Day 0.

Fertilization was assessed 16–18 h after ICSI by the presence of two pronuclei (2PN). Normally fertilized zygotes were cultured individually in sequential medium (Vita, Shenzhen, China). The following outcomes were assessed: maturation rate at 24 h, maturation rate at 48 h, fertilization rate, cleavage rate.

### 2.6 Porcine Oocyte Collection and In Vitro Maturation

All procedures followed the Animal Research Protocol approved by Nanjing Agricultural University. Ovaries from prepubertal gilts were recovered at a local abattoir, held in 0.9% saline at 38°C, and delivered to the laboratory within 2 h. Following two DPBS washes, 3–6 mm antral follicles were punctured with 20‐gauge needles; only COCs showing intact, compact cumulus investment and homogeneous ooplasm were kept. Basal maturation medium was TCM‐199 fortified with 75 μg mL^−1^ penicillin, 50 μg mL^−1^ streptomycin, 0.5 μg mL^−1^ FSH, 0.5 μg mL^−1^ LH, 10 ng mL^−1^ EGF and 0.57 mM cysteine. Groups of 25–30 COCs were matured in 500 μL droplets under mineral oil at 38.5°C, 5% CO_2_, for 27 or 44 h. Cumulus cells were then removed by a 5‐min exposure to 0.1% hyaluronidase at 38.5°C and gentle pipetting; denuded oocytes were washed 3–4 times and examined under an inverted microscope (×200) for polar‐body extrusion.

### 2.7 Elamipretide Treatment

In the in vivo experimental design, elamipretide powder was dissolved in normal saline to formulate a 100 mM solution, which was then aliquoted and stored at −20°C. Prior to use, the solution was thawed at room temperature and administered to aged mice via intraperitoneal injection at three different dosages: 3, 5, and 10 mg/kg/day. The efficacy of these dosages was evaluated, and a dose of 5 mg/kg/day was identified as the optimal therapeutic dose. To further optimize the treatment protocol, three different treatment durations—3, 7, and 10 days—were tested. Ultimately, a 7‐day treatment duration was determined to be the most effective therapeutic regimen.

### 2.8 Immunofluorescent Staining and Confocal Microscopy

At least 30 oocytes were initially fixed in a 4% paraformaldehyde solution in PBS (pH 7.4) for 30 min. Following fixation, the oocytes were permeabilized using 0.5% Triton‐X‐100 for 20 min at room temperature. After permeabilization, the oocytes were blocked with 1% bovine serum albumin (BSA) and subsequently incubated with primary antibodies, including γ‐tubulin (1:200), acetylated tubulin (1:400), Rab10 (1:500), GRP78 (1:200), LC3 (1:100), and anti‐α‐tubulin‐FITC (1:100), overnight at 4°C. After the incubation period, the oocytes were transferred to a washing solution composed of 0.1% Tween 20 and 0.01% Triton X‐100 in PBS and subjected to three washing cycles. Subsequently, the oocytes were labeled with Alexa Fluor 488 or Alexa Fluor 594‐conjugated secondary antibodies at a dilution of 1:100. To visualize F‐actin via phalloidin staining, the oocytes were subjected to incubation with phalloidin‐TRITC at room temperature for a duration of 1 h. Subsequent to this step, the chromosomes within the oocytes were stained using Hoechst 33342 for a period of 10 min. The oocytes were subsequently affixed to glass slides and analyzed using a confocal laser scanning microscope fitted with a 40× water immersion objective lens (Zeiss LSM 700 META, Germany). Moreover, live cell fluorescence staining was employed to evaluate the distribution of mitochondria with Mito Tracker Red CMXRos (Invitrogen, USA), MMP (Mito Probe JC‐1 Assay kit, Invitrogen, USA), and calcium level (Fluor 4.AM, Beyotime Biotechnology, Nantong, China), endoplasmic reticulum (Beyotime Biotechnology, Nantong, China), and lysosome (Invitrogen, Eugene, OR, USA). Prior to transfer into M2 medium supplemented with fluorescent probes, oocytes were cultured to reach the desired meiotic stage. Following a 30‐min incubation at 37°C under 5% CO_2_ conditions, the oocytes were immediately subjected to confocal microscopy imaging.

### 2.9 mtDNA Copy‐Number Assessment

Fifty oocytes per group were pooled and processed with the Bioengineering mitochondrial DNA isolation kit (B518749‐0050). Relative mtDNA abundance was determined by qPCR, normalizing the mitochondrial amplicon to the nuclear β‐actin sequence. Primer pairs (synthesized by Genewiz) were: β‐actin: 5′‐TGT GAC GTT GAC ATC CGT AA‐3′ and 5′‐GCT AGG AGC CAG AGC AGT AA‐3′; mitochondrial target: 5′‐CCA ATA CGC CCT TTA ACA AC‐3′ and 5′‐GCT AGT GTG AGT GAT AGG GTA G‐3′.

### 2.10 ATP Quantification

Thirty oocytes per group were lysed and processed with the Sigma bioluminescent ATP kit (Cat# 1003493051). After brief permeabilization with the supplied, pre‐diluted ATP‐release reagent, samples were combined with luciferase substrate and immediately read on a Tecan Spark multimode reader; emitted light was converted to relative ATP concentrations.

### 2.11 Western Blotting

Around 50 mouse oocytes were solubilized in NuPAGE LDS sample buffer, heated (10 min, 100°C), and kept at −20°C until use. Equal volumes were loaded on 10% SDS‐polyacrylamide gels and resolved at 160 V for 70 min. Proteins were electro‐transferred to PVDF membranes (Millipore, Billerica, MA) at 20 V for 90 min. After blocking with fast blocking buffer (0.5 h, RT), membranes were probed overnight at 4°C with primary antibodies (rabbit anti‐DRP1, rabbit anti‐α‐tubulin, mouse anti‐β‐actin, anti‐GM130, anti‐GRP78, and anti‐acetyl‐tubulin, anti‐LC3, anti‐ROCK1, all 1:1000). Following five TBST washes (10 min each), HRP‐conjugated goat anti‐rabbit or anti‐mouse IgG (1:2000) was applied for 1 h at RT. Signals were developed with YEASEN high‐sig/super‐sig ECL (1:10) and quantified by ImageJ.

### 2.12 Real‐Time Quantitative PCR Analysis

Real‐time quantitative PCR (qPCR) was employed to assess the mRNA expression level of genes. Total RNA was extracted from a pool of 30 oocytes utilizing the Dynabeads mRNA DIRECT kit (Invitrogen Dynal AS, Norway). Following RNA isolation, first‐strand cDNA synthesis was conducted using the PrimeScript RT Master Mix (Takara, Japan) with the thermal cycling conditions of 37°C for 15 min, 85°C for 5 s, and a final hold at 4°C. The primer sequences used for amplifying the cDNA fragments of the genes are listed in the table in the attachment. The PCR protocol was carried out under the following conditions: an initial denaturation at 95°C for 30 s, succeeded by 40 cycles of quantitative PCR (qPCR) amplification (95°C for 5 s and 60°C for 30 s), and concluded with a melt‐curve analysis comprising 95°C for 5 s, 60°C for 60 s, and a final step at 95°C for 1 s, with the reaction maintained at 4°C. The primers are listed in Table S1.

### 2.13 Statistical Analysis

For each experimental analysis, a minimum of three biological replicates was performed to ensure reliability. The mean values and standard error of the mean (SEM) were calculated. Statistical significance between the three groups was evaluated using ordinary One‐Way ANOVA. The statistical analyses were carried out within GraphPad Prism 5 software. Results were deemed statistically significant when the *p*‐value was less than 0.05 (indicated by *) and highly significant when the *p*‐value was less than 0.01 (indicated by **).

## 3 Results

### 3.1 Short‐Term Elamipretide Treatment Improves Fertility of Aged Mice

An optimal therapeutic protocol for elamipretide in aged mice was initially established, with body weight and ovarian status systematically documented in both young and aged mice (Figure S1A). As shown in Figure 1A, daily intraperitoneal elamipretide was administered to aged mice for 3, 7 or 10 days, with age‐matched and young controls receiving equivalent saline. Upon completion, mice were either naturally mated for fertility evaluation or super‐ovulated with PMSG/hCG to harvest oocytes for subsequent assays. We assessed daily doses of 3, 5, and 10 mg/kg in 10‐month‐old aged mice and collected MII oocytes from the ampulla. The 5 mg/kg regimen produced the most optimal efficacy in increasing the number of ovulations (Figure 1B). In Figure 1C, using 5 mg/kg/day, we treated 8‐, 10‐, and 12‐month‐old mice for 3, 7 or 10 days and then assessed fertility. The 7‐day regimen improved reproductive performance in all age groups, with the most pronounced gain observed in 10‐month‐old mice. The beneficial effect of elamipretide on fertility was limited to a short‐term window of only 1 month (Figure S1B). Consequently, the subsequent studies were therefore performed in 10‐month‐old mice subjected to the 7‐day elamipretide protocol. Ovarian sections likewise showed that elamipretide restored the number of dominant follicles and rejuvenated the aged ovary (Figure 1D). Elamipretide markedly elevated the fertilization rate in aged oocytes, confirming a substantial recovery of their fertilization potential (Figure 1E). This was also consistent with its role on repairing cortical granule trafficking (Figure S1C). Following fertilization, aged oocytes exhibited a reduced 2‐cell rate and a markedly lower 4‐cell rate than young controls, while elamipretide effectively reversed 2‐ to 4‐cell progression, restoring early embryonic development (Figure 1F). Therefore, elamipretide restores the dominant‐follicle reserve and improves fertility in aged mice.

**FIGURE 1:**
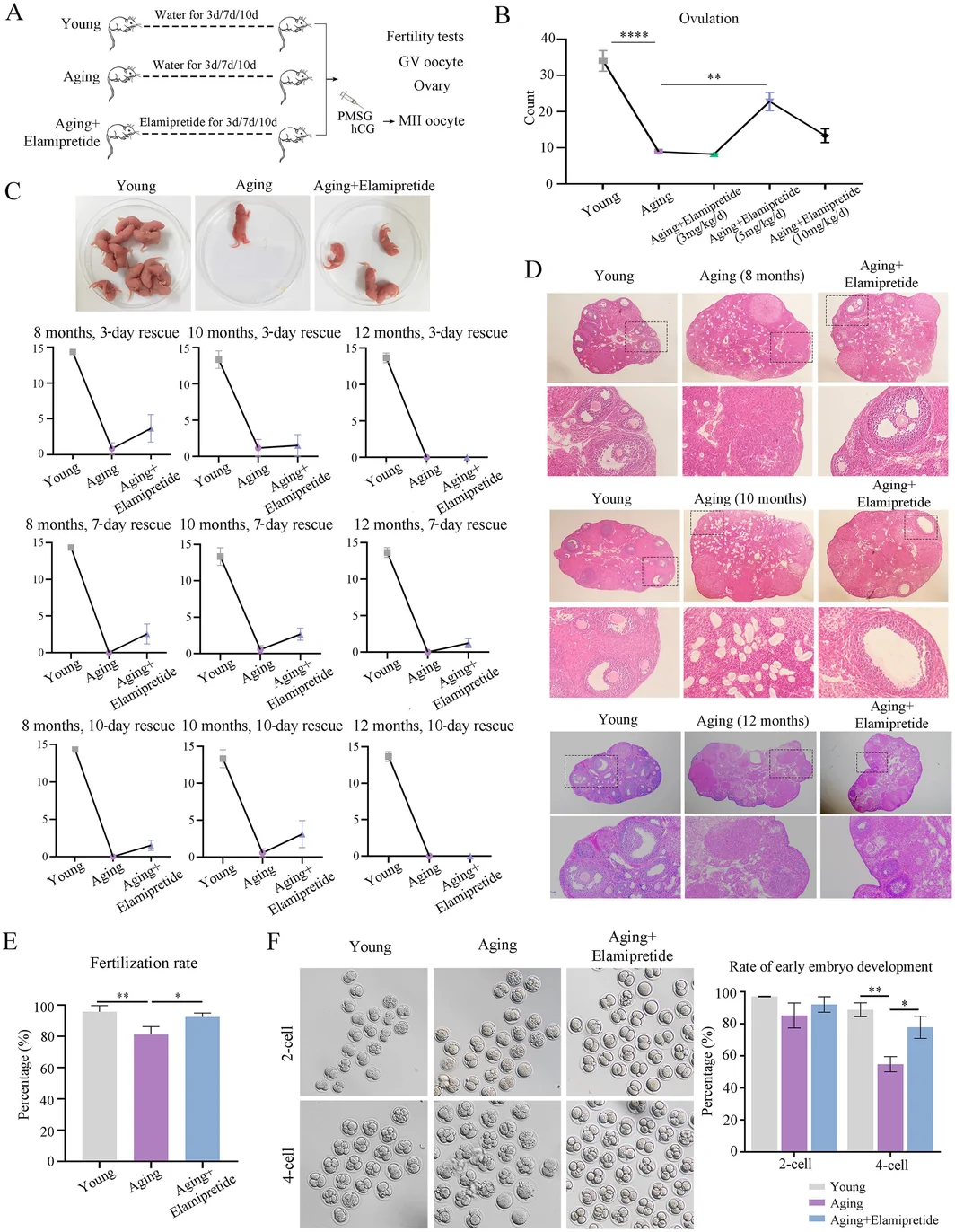
Short‐term elamipretide treatment boosts fertility in aged mice. (A) Treatment regimen of elamipretide in aged mice. (B) Dose‐escalation study to determine the optimal elamipretide regimen. (C) Fertility assessment across varying treatment durations. (D) Ovarian sections from 8‐, 10‐ and 12‐month‐old mice after 7‐day elamipretide treatment showed a marked increase in high quality of follicles. (E) Elamipretide restored fertilization rates after in vitro fertilization. (F) Elamipretide effectively rescued the 2‐ to 4‐cell embryo transition. *p < 0.05, **p < 0.01, ****p < 0.0001.

### 3.2 Elamipretide Reshapes Ovarian Vitamin B6 Metabolism in Aged Mice

We performed metabolomics to examine the metabolism change after elamipretide treatment. First, all detected metabolites were clustered based on their temporal dynamics, yielding 12 distinct expression patterns (Figure 2A). In Figure 2B, compared with the young group, aged ovaries exhibited 250 differentially accumulated metabolites (122 up, 128 down), whereas elamipretide treatment shifted 30 metabolites relative to the aged group (12 up, 18 down). We performed principal‐component analysis on the complete metabolome and the differential metabolite subset, uncovering their overall distribution patterns and key discriminating dimensions (Figure 2B). The heat map revealed an age‐dependent metabolite signature that elamipretide restored to a young‐like profile, and the Venn diagram revealed 11 differential metabolites shared between the two comparison groups (Figure 2C). Figure 2D illustrated that metabolite abundance was markedly reduced in aged mice yet effectively restored to youthful abundance following elamipretide treatment. We performed GO‐based enrichment clustering on the filtered metabolome and plotted the top 14 most over‐represented metabolic categories as a bar chart (Figure 2E). We then generated correlation networks for the differential metabolites identified in young vs. aging and aging vs. aging+elamipretide comparisons to visualize their interrelationships and regulatory hubs (Figure 2F). We further mapped the differential metabolites to KEGG pathways, extracting the top 10 enriched routes for each pairwise comparison, with the vitamin B6 pathway showing a pronounced alteration (Figure 2G). Additionally, we constructed a heat map showing the relative abundance of the 11 shared metabolites across the different groups (Figure 2H). We next selected the most dramatically altered metabolites, LAC (L‐Acetylcarnitine) and AVA (Aminovaleric acid). Individual or in combined treatment with them failed to restore first polar body extrusion in aged oocytes, whereas VB6 supplementation fully reversed the age‐associated decline (Figure 2I). Therefore, elamipretide may enhance the maturational quality of aged oocytes by modulating vitamin B6 metabolism.

**FIGURE 2:**
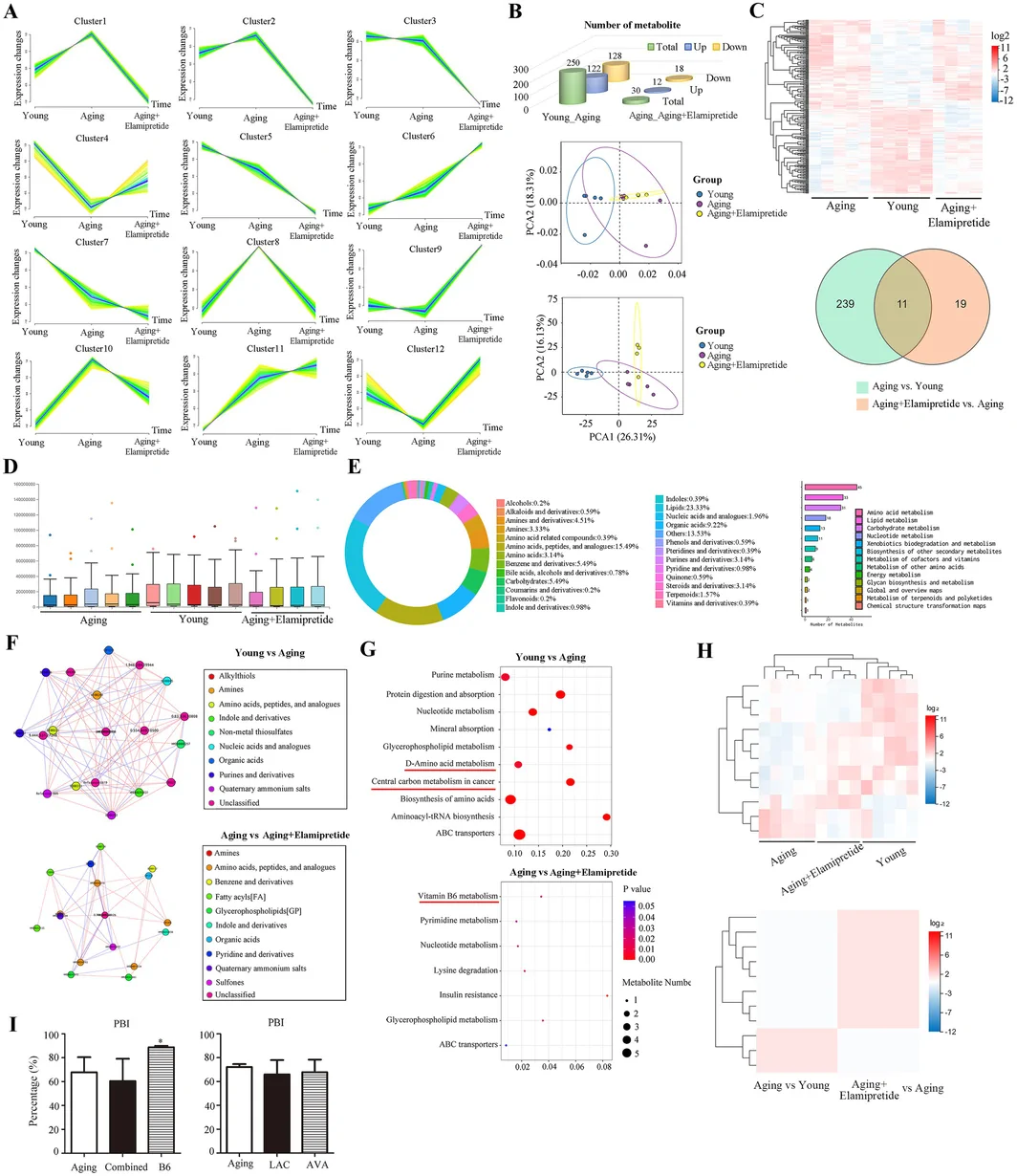
Elamipretide reshapes ovarian Vitamin B6 metabolism in aged mice. (A) Metabolite expression profiles across sample groups were clustered, grouping metabolites with similar expression patterns into the same cluster. (B) The number of up‐ and down‐regulated differential metabolites was tallied, and PCA dimensionality reduction was used to visualize the inter‐group separation trend. (C) Hierarchial clustering of differential metabolites directly revealed their expression patterns across the three sample groups, 11 differential metabolites were shared between the two comparison groups. (D) Global abundance profiling of all detected metabolites across every group. (E) Circular chart of metabolite categories and bar chart of metabolic pathway classification. (F) Correlation network map of differential metabolites. (G) Bubble plot of metabolic pathway enrichment analysis. (H) Hierarchical clustering of the 11 shared differential metabolites. (I) LAC, AVA, and vitamin B6 supplementation on the rejuvenation of aged oocyte developmental competence. *p < 0.05.

### 3.3 Elamipretide Alters General Transcript Levels in Mouse Aged Oocytes

We next collected the oocytes for transcriptome analysis, with 15 oocytes each group. In the aging group compared with the young group, there was a total of 829 genes changed, with 420 upregulated and 409 genes downregulated. While in the elamipretide treatment group compared with the aging group, of the 2081 differentially expressed genes, 965 were upregulated and 1116 were downregulated (Figure 3A). In Figure 3B, it was showed that between these two sets of differentially expressed genes, 518 genes were commonly shared. The volcano plot displays the fold changes of differentially expressed genes and distinguishes the upregulated from the downregulated. The heatmap clusters differentially expressed genes, allowing us to visualize the overall expression patterns across the young, the aging, and the aging + elamipretide groups (Figure 3C). Then we performed GO enrichment analysis on the significantly differentially expressed genes in Figure 3D. We found that elamipretide treatment alleviates alterations in membrane‐bound organelles and lipid transport and metabolism processes. Subsequent KEGG analysis of the differentially expressed genes reveals that the VEGF signaling pathway is markedly altered in oocytes from aged mice compared with the young, and the VEGF pathway expression is reversed in elamipretide‐treated mouse oocytes (Figure 3E). Therefore, elamipretide may modulate the maturation quality of aged oocytes via the VEGF pathway.

**FIGURE 3:**
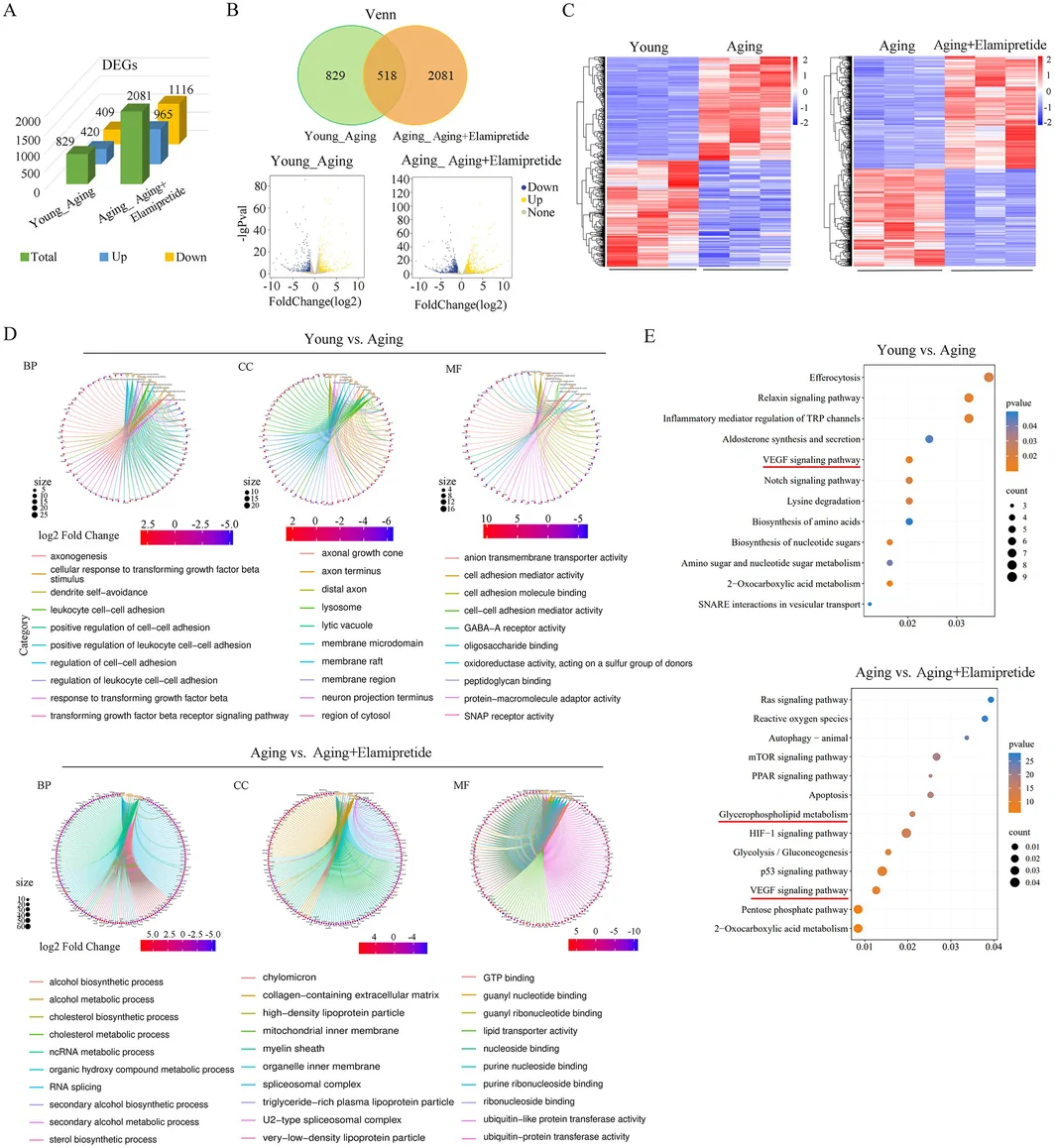
Elamipretide alters general transcript levels in mouse aged oocytes. (A) Differential gene expression showed bidirectional regulation, with up‐ and down‐regulated genes clearly segregated. (B) Venn and volcano plots of differentially expressed genes revealed 518 shared genes. (C) Heat map of differentially expressed genes. (D) Chord diagram of GO enrichment clusters for differentially expressed genes. (E) Top 12 KEGG pathways enriched by differentially expressed genes shown in a bubble plot.

### 3.4 Elamipretide Promotes Cytoskeletal Dynamics and Spindle Migration of Aged Oocytes

Using GO analysis, we identified cytoskeleton‐related biological processes rescued by elamipretide versus aged oocytes (Figure 4A). In the Figure 4B, we first examined spindle assembly and chromosome alignment, which showed that aged oocytes displayed aberrant spindles and scattered chromosomes, whereas elamipretide reduced the frequency of these defects. γ‐tubulin staining revealed that aged oocytes lost γ‐tubulin foci at the poles, and elamipretide restored them (Figure 4C). Acetylated tubulin is commonly used as a marker of microtubule stability. Our results showed that aging decreased the level of tubulin acetylation in mouse oocytes, indicating that aging compromises microtubule stability, whereas supplementation with elamipretide restored tubulin acetylation levels (Figure 4D). Additionally, we assessed whether actin‐network organization was compromised. Cortical actin assembly remained intact in aged oocytes, and elamipretide treatment did not alter it (Figure 4E). Notably, cytoplasmic actin‐network assembly was disrupted by aging, and this decline was effectively reversed by elamipretide supplementation (Figure 4F). Subsequently, we detected the expression levels of related proteins via western blot analysis. The results demonstrated that Arp2 and ROCK1, the molecules related to actin assembly, were significantly downregulated in aged oocytes, and treatment with elamipretide failed to reverse the decreased expression of Arp2 but restored ROCK1/2 (Figure 4G). Thus, elamipretide sustains spindle assembly and migration in aged oocytes by elevating microtubule acetylation and promoting cytoplasmic actin nucleation.

**FIGURE 4:**
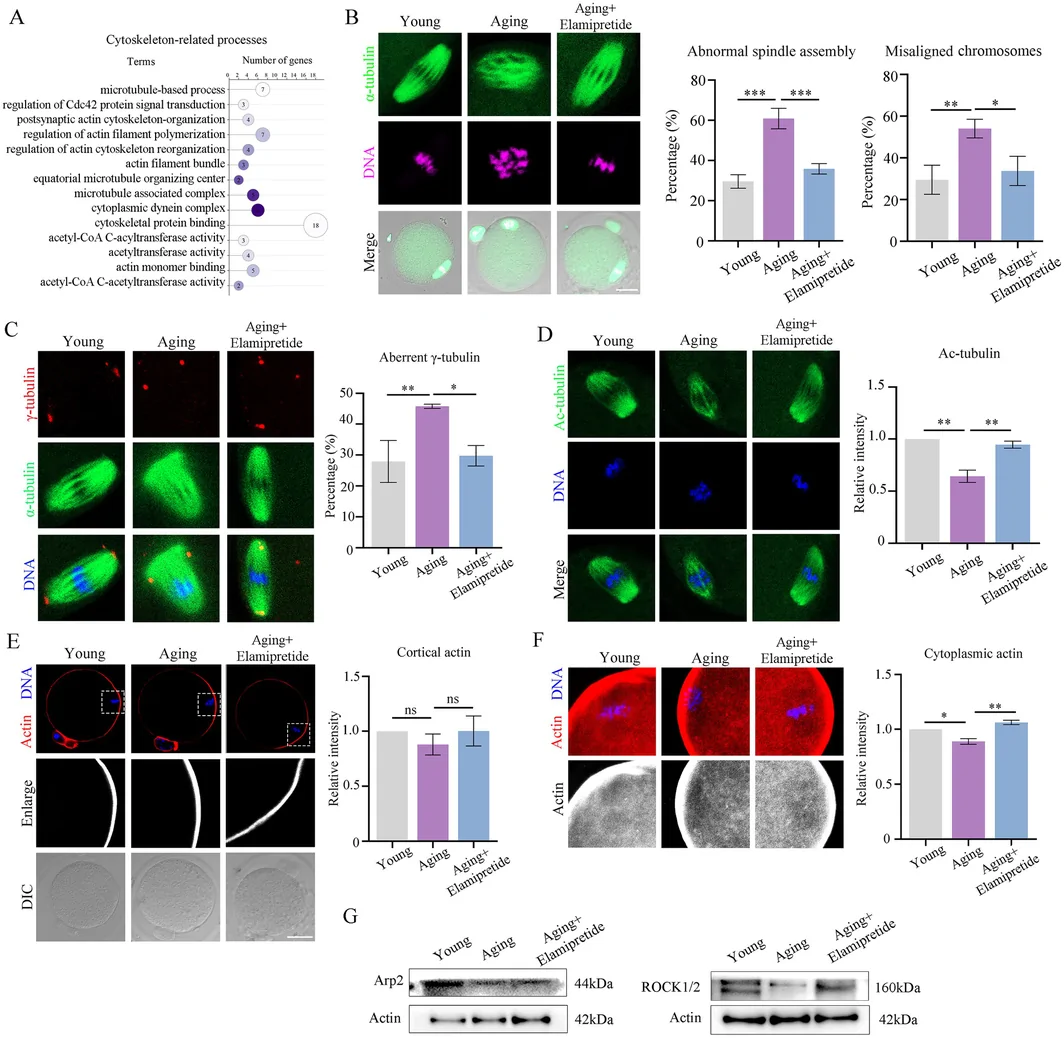
Elamipretide promotes cytoskeletal assembly and spindle migration in mouse aged oocytes. (A) GO classification of cytoskeleton‐related processes improved by elamipretide treatment in aged oocytes. (B) Elamipretide restored orderly spindle assembly and corrects chromosome alignment. Green, α‐tubulin; purple, DNA; scale bar, 20 μm. (C) Elamipretide re‐established the proper localization of γ‐tubulin. Red, γ‐tubulin; green, α‐tubulin; blue, DNA; scale bar, 20 μm. (D) Elamipretide boosted acetylated tubulin levels, stabilizing the meiotic spindle in aged oocytes. Green, α‐tubulin; blue, DNA; scale bar, 20 μm. (E) Cortical F‐actin remained unchanged in aged oocytes. (F) The cytoplasmic actin was significantly reduced in aged oocytes, and elamipretide fully restored cytoplasmic actin levels. Red, Actin; blue, DNA; scale bar, 20 μm. *p < 0.05, **p < 0.01, ***p < 0.001, and ns, no significant difference. (G) The protein level of Arp2 and ROCK1/2 after elamipretide supplement in mouse aged oocytes.

### 3.5 Elamipretide Alleviates Mitochondria‐Driven Oxidative Stress in Aged Oocytes

Oocyte maturation extends beyond nuclear progression to include cytoplasmic maturation, with mitochondrial rearrangement as its pivotal event. From the transcriptome analysis, we first extracted the nine most significantly enriched GO pathways distinguishing the aging group from the aging+elamipretide group. In Figure 5A, differentially expressed genes were chiefly enriched for inner mitochondrial membrane organization, mitochondrial metabolism, and oxidative‐stress response. We selected a panel of mitochondria‐related key genes and quantified the mRNA levels by qPCR; elamipretide fully reversed the age‐associated transcriptional changes (Figure 5B). Figure 5C showed that aging displaced oocyte mitochondria from the peri‐spindle domain and drove their condensation into cytoplasmic clusters, while elamipretide restored mitochondrial positioning. We found that elamipretide normalized the supraphysiological ATP synthesis to young levels and partially reinstated mitochondrial DNA copy number (Figure 5D). TMRE is a fluorescent probe for quantifying MMP, and we found that MMP declined in aged oocytes, likely accounting for the reduced ATP production, and elamipretide treatment restored potential to a higher level (Figure 5E). We further examined the expression levels of mitochondrial fission proteins INF2 and DRP1. GAPDH was used as the control, and the results showed that INF2 expression was significantly decreased in aging oocytes, and elamipretide treatment failed to restore its expression level (Figure 5F). In contrast, elamipretide significantly upregulated DRP1 expression in aged oocytes (Figure 5G). Mitochondrial depolarization commonly signals intensified oxidative stress and ROS burst, so we next quantified the oxidative‐stress burden in oocytes. We profiled a panel of oxidative‐stress‐related genes and found that elamipretide partially reversed their age‐induced overexpression (Figure 5H). While elamipretide markedly restored redox homeostasis in aged oocytes, showing with reduced ROS levels (Figure 5I). Additionally, we examined oxidative‐stress‐induced apoptosis in aged oocytes. Elamipretide reversed the transcriptional changes of several apoptosis‐related genes (Figure 5J), but fluorescence staining showed that it only partly reduced the oocytes with positive apoptosis signals caused by aging with no significant difference (Figure 5K). The findings indicate that elamipretide can restore oxidative stress by improving mitochondrial distribution and function in aged oocytes.

**FIGURE 5:**
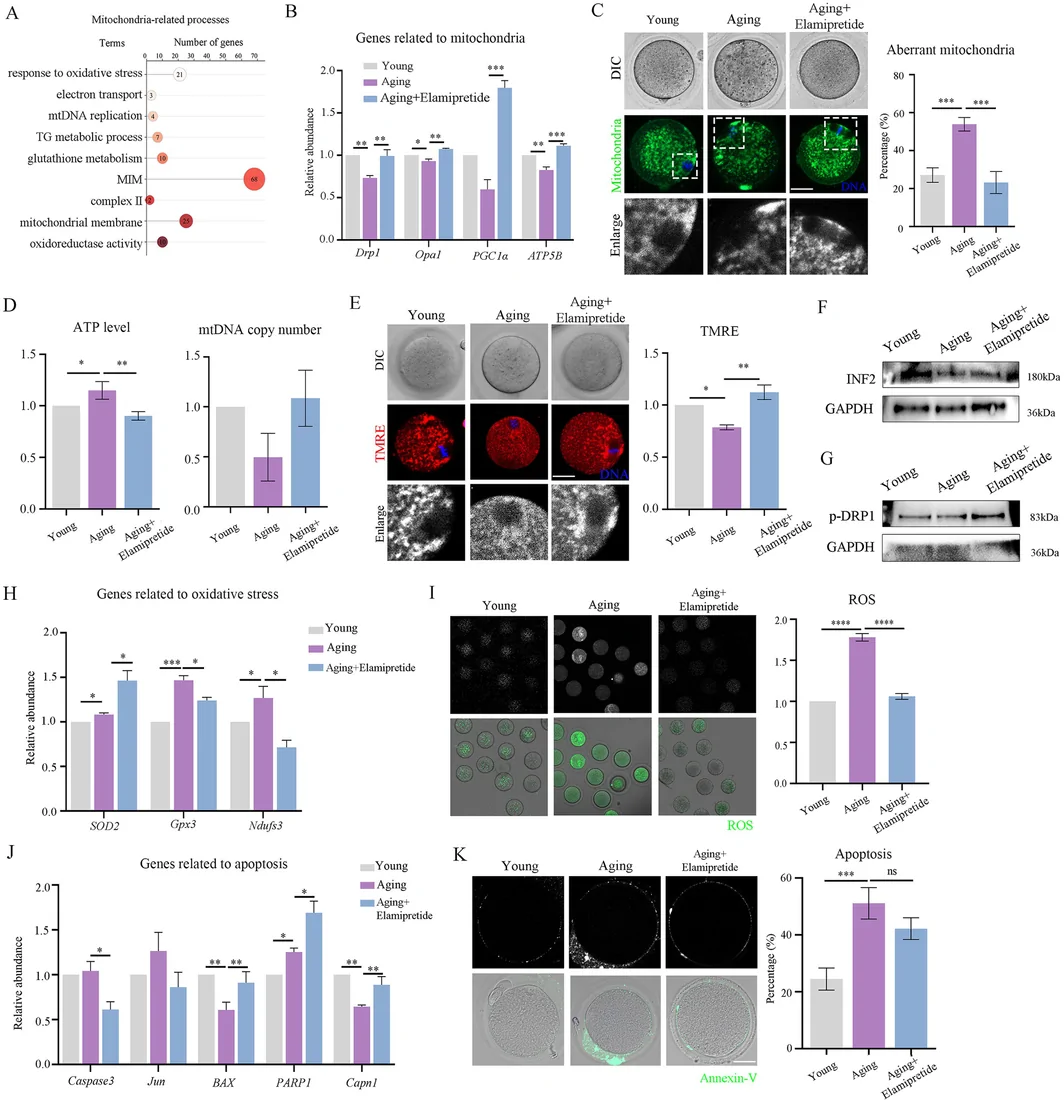
Elamipretide alleviates mitochondria‐driven oxidative stress in mouse aged oocytes. (A) GO clusters related to mitochondrial metabolism identified between aging + elamipretide versus aged oocytes. (B) Quantification of mRNA levels for mitochondria‐related functional genes. (C) Elamipretide corrected the aberrant peri‐spindle distribution of mitochondria in mouse aged oocytes. Green, mitochondria; blue, DNA; scale bar, 20 μm. (D) Elamipretide restored overly elevated ATP to physiological levels and simultaneously boosted mitochondrial number. (E) TMRE reported mitochondrial function, and elamipretide ameliorated mitochondrial dysfunction by elevating mitochondrial membrane potential. Red, TMRE; blue, DNA; scale bar, 20 μm. (F) Protein expression level of INF2 after elamipretide supplement in mouse aged oocytes. (G) Protein expression level of p‐DRP1 after elamipretide supplement in mouse aged oocytes. (H) Changes in mRNA expression of oxidative‐stress‐related genes. (I) Elamipretide brings excessive oxidative stress in aged oocytes back to youthful levels. Green, ROS; scale bar, 20 μm. (J) Alterations in mRNA levels of apoptosis‐related genes. (K) Elamipretide partly reduced oxidative‐stress‐induced apoptosis in aged oocytes with no significance. Green, Annexin‐V; scale bar, 20 μm. *p < 0.05, **p < 0.01, ***p < 0.001, ****p < 0.0001, and ns, no significant difference.

### 3.6 Elamipretide Restores Organelle‐Associated Processes in Aged Oocytes

Cytoplasmic maturation also encompasses the remodeling and functional maturation of organelles such as the endoplasmic reticulum and Golgi apparatus. By screening membrane‐organelle pathways, we found that ER‐, Golgi‐ and lysosome‐related processes were all perturbed (Figure 6A). To assess the impact of elamipretide on Golgi function, we mapped Rab10 localization in oocytes and we found that elamipretide fully reversed the age‐dependent decline in Rab10 expression, restoring it to normal levels (Figure 6B). In Figure 6C, analysis of Golgi‐related transcripts revealed that elamipretide rescued the age‐dependent decline in the functional marker GM130, restoring its expression to normal levels. β‐actin was used as the control for western blots detecting cytoskeleton‐associated and cytoplasmic proteins, and our results further confirmed that elamipretide effectively restored the expression levels of GM130 and Rab10 in aged oocytes (Figure 6D). The ER also plays a pivotal role in oocyte cytoplasmic maturation. With aging, ER strands abandoned their spindle‐adjacent stronghold and scattered evenly throughout the cytoplasm. And elamipretide returned them to the subcortical spindle zone (Figure 6E). Further analysis of the ER stress marker GRP78 revealed that elamipretide effectively reversed its age‐dependent decline, indicating that the peptide rescued insufficient ER stress adaptation (Figure 6F). Furthermore, elamipretide also effectively restored the decreased expression of GRP78 in aged oocytes, thereby improving the endoplasmic reticulum stress response in oocytes (Figure 6G). Following elamipretide intervention, transcript levels of the ER‐related genes GRP78, CHOP and ATF4 returned to normal, further confirming the restorative effect on ER function (Figure 6H). Additionally, lysosome‐mediated autophagic activity constitutes an equally indispensable facet of cytoplasmic maturation. In aged oocytes autophagic vacuoles accumulated abnormally and lysosomes lost their normal distribution, and elamipretide restored the even cytoplasmic pattern (Figure 6I). In senescent oocytes LC3‐positive vesicles were fewer and scattered; elamipretide treatment amplified LC3 fluorescence and reinstated autophagic activity (Figure 6J). Western blot analysis demonstrated that the expression level of LC3 was decreased in aged oocytes, and supplementation with elamipretide restored its expression (Figure 6K). Transcript profiling of autophagy genes showed elamipretide returned their mRNA abundance to the normal range (Figure 6L). The results demonstrate that elamipretide orchestrates organelle repositioning and concomitantly restores protein synthesis and autophagic flux in aged oocytes.

**FIGURE 6:**
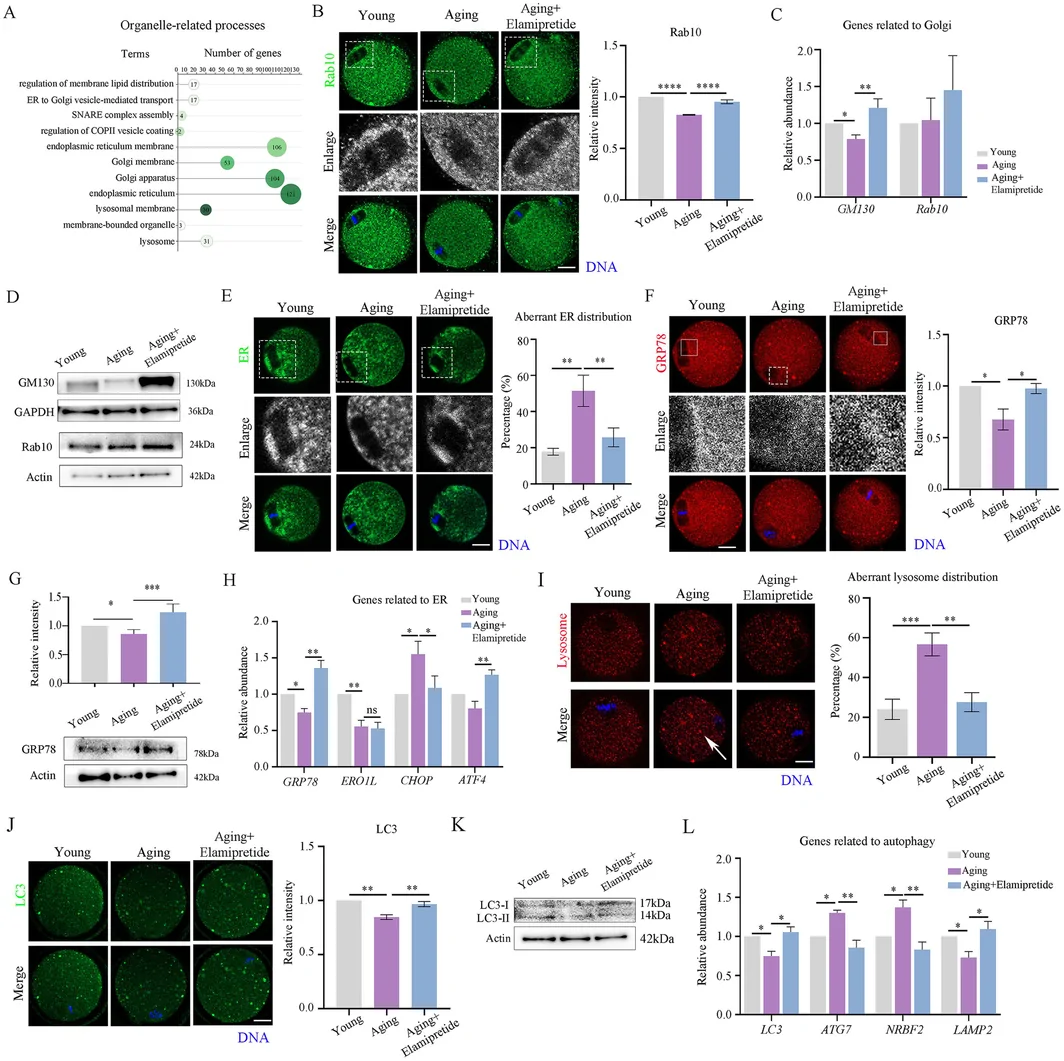
Elamipretide restores organelle‐associated processes in mouse aged oocytes. (A) GO analysis of organelle‐related processes after elamipretide supplement compared with the aging group. (B) The fluorescence intensity analysis of the Golgi functional protein Rab10 after elamipretide treatment. Green, Rab10; blue, DNA; scale bar, 20 μm. (C) Detection of mRNA expression levels of Golgi‐associated genes GM130 and Rab10. (D) Protein expression level of GM130 and Rab10 in the elamipretide group compared with the young and aging groups. (E) Elamipretide restored the loss of ER localization around the spindle in aged oocytes. Green, ER; blue, DNA; scale bar, 20 μm. (F) The ER stress protein GRP78 was aberrantly reduced in aged oocytes, and elamipretide restored it to normal levels. Red, GRP78; blue, DNA; scale bar, 20 μm. (G) Protein expression level of GRP78 after elamipretide supplement compared with young and aging groups. (H) Changes in the mRNA expression levels of ER‐related genes after elamipretide supplement. (I) Elamipretide alleviated the excessive accumulation of lysosomal autophagic vesicles in oocytes caused by aging. Red, lysosome; blue, DNA; scale bar, 20 μm. (J) elamipretide recovered the abnormal reduced LC3 expression levels in aged oocytes. Green, LC3; blue, DNA; scale bar, 20 μm. (K) Protein expression level of LC3 after elamipretide supplement compared with young and aging groups. (L) The mRNA expression levels of autophagy‐related genes were significantly improved after elamipretide treatment. *p < 0.05, **p < 0.01, ***p < 0.001, ****p < 0.0001, and ns, no significant difference.

### 3.7 Elamipretide Restores Aged Oocyte Quality via VEGF‐Dependent Pathway

As shown in Figure 7A, transcriptome profiling identified VEGF‐pathway genes with significant differential expression, and a heatmap was generated. We then validated these genes by qRT‐PCR, confirming the expression trends observed in the transcriptome data (Figure 7B). Western blot analysis further revealed that aging elevated VEGF‐pathway protein levels, and elamipretide treatment effectively regained them to baseline (Figure 7C). We first interrogated the PI3K/AKT axis with LY294002 and Wortmannin. In Figure S2A, 4 μM LY294002 was selected as the working concentration; hyper‐assembled cytoplasmic microfilaments were normalized, yet cortical F‐actin remained unchanged (Figure S2B). As PI3K/AKT is a canonical apoptosis pathway, we also assessed Annexin‐V level. However, no protective effect was evident (Figure S2C). Likewise, Wortmannin failed to alleviate oxidative stress in aged oocytes (Figure S2D). Hence, PI3K/AKT is unlikely to serve as a downstream effector of elamipretide in aged oocytes. To examine whether elamipretide improves oocyte quality via VEGF pathway, we treated aged oocytes with PTC299 to inhibit VEGF‐A transcriptional activity. Concentration‐response analysis identified 5 μM as the most effective dose for rescuing oocyte developmental quality (Figure 7D). It was demonstrated that PTC299 reversed the age‐dependent mitochondrial displacement, re‐establishing the uniform peri‐spindle distribution characteristic (Figure 7E). In aged oocytes, VEGF‐A transcriptional inhibition by PTC299 also attenuated the mitochondria‐associated ROS surge (Figure 7F, Figure S2E). And the aberrant apoptosis triggered by oxidative stress was also ameliorated by PTC299 (Figure 7G). Furthermore, PTC299‐mediated inhibition of VEGF translation repaired the spindle assembly/migration defects and chromosome misalignment of aged oocytes (Figure 7H). Actin filament assembly, which is essential for spindle migration, was likewise restored to normal levels (Figure S2F). Together, the data position VEGF‐A as a requisite node through which elamipretide restores developmental competence in aged oocytes.

**FIGURE 7:**
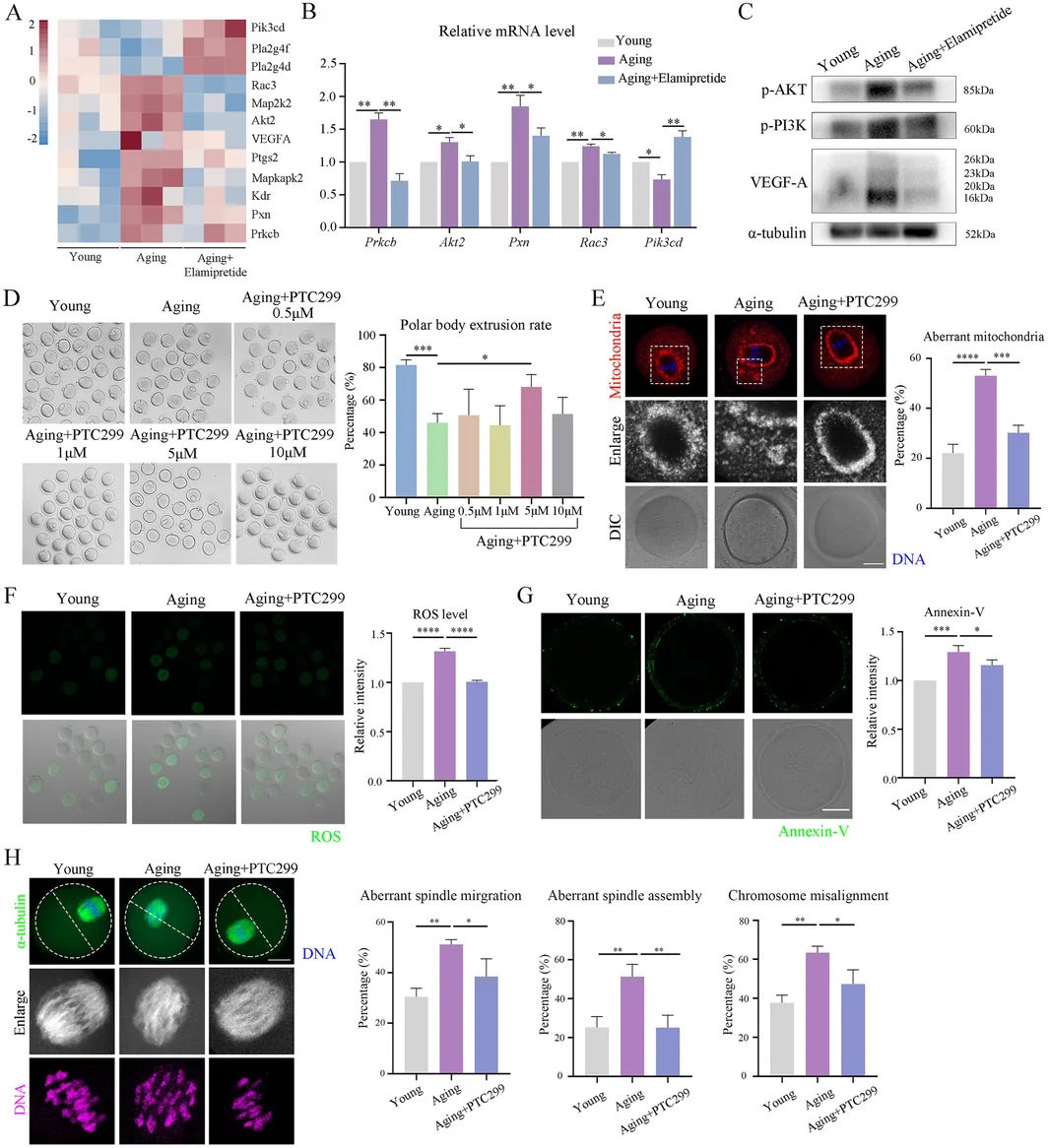
Elamipretide restores mouse aged oocyte quality via VEGF‐dependent pathway. (A) Heatmap of differentially expressed genes in the VEGF pathway identified by transcriptome analysis. (B) qRT‐PCR was used to profile pathway gene expression, thereby confirming the reliability of the transcriptome results. (C) Western blot was used to detect the expression levels of VEGF pathway proteins in the three groups. (D) A concentration‐gradient screen of VEGF inhibitors identified 5 μM as the optimal treatment concentration for the polar body extrusion in mouse aged oocytes. (E) PTC299 reversed the age‐induced mis‐localization of mitochondria in mouse oocytes. Red, mitochondria; blue, DNA; scale bar, 20 μm. (F) PTC299 curbed the oxidative‐stress surge elicited by aging in mouse oocytes. Green, ROS; scale bar, 20 μm. (G) Inhibiting VEGF translation activity with PTC299 decreased oocyte early apoptosis in aged mice. Green, Annexin‐V; scale bar, 20 μm. (H) PTC299 restored defective spindle assembly and migration and corrected chromosome misalignment in mouse aged oocytes. Green, α‐tubulin; purple, DNA; scale bar, 20 μm. *p < 0.05, **p < 0.01, ***p < 0.001, ****p < 0.0001.

### 3.8 Elamipretide Treatment Improves Human Oocyte Maturation, Fertilization and Cleavage

A total of 39 patients were enrolled. In the advanced‐age group (≥ 35 years), three patients (mean age 38.45 ± 3.03 years) contributed 54 GV oocytes, which were randomly assigned to the experimental (*n* = 29) or control (*n* = 25) group. In the younger group (< 35 years), 19 patients (mean age 30.32 ± 2.97 years) contributed 75 GV oocytes, assigned to the experimental (*n* = 37) or control (*n* = 38) group. We first tested the effects of elamipretide on human oocytes of both aged women (> 36 years). We found that supplementation of elamipretide in the in vitro maturation culture system of oocytes from AMA women, promoted oocyte developmental progression, and effectively ensured successful first polar body extrusion (Figure 8A). As shown in Figure 8B, in vitro culture of human aged oocytes with elamipretide improved the maturation with both 24 h culture and 48 h culture, while there is a increase for the fertilization rate and cleavage rate after ICIS with MII oocytes. Similarly, elamipretide supplement on the oocytes of young women also showed higher maturation rate and cleavage rate. These data suggested that elamipretide treatment could improve oocyte maturation and developmental competence in human.

**FIGURE 8:**
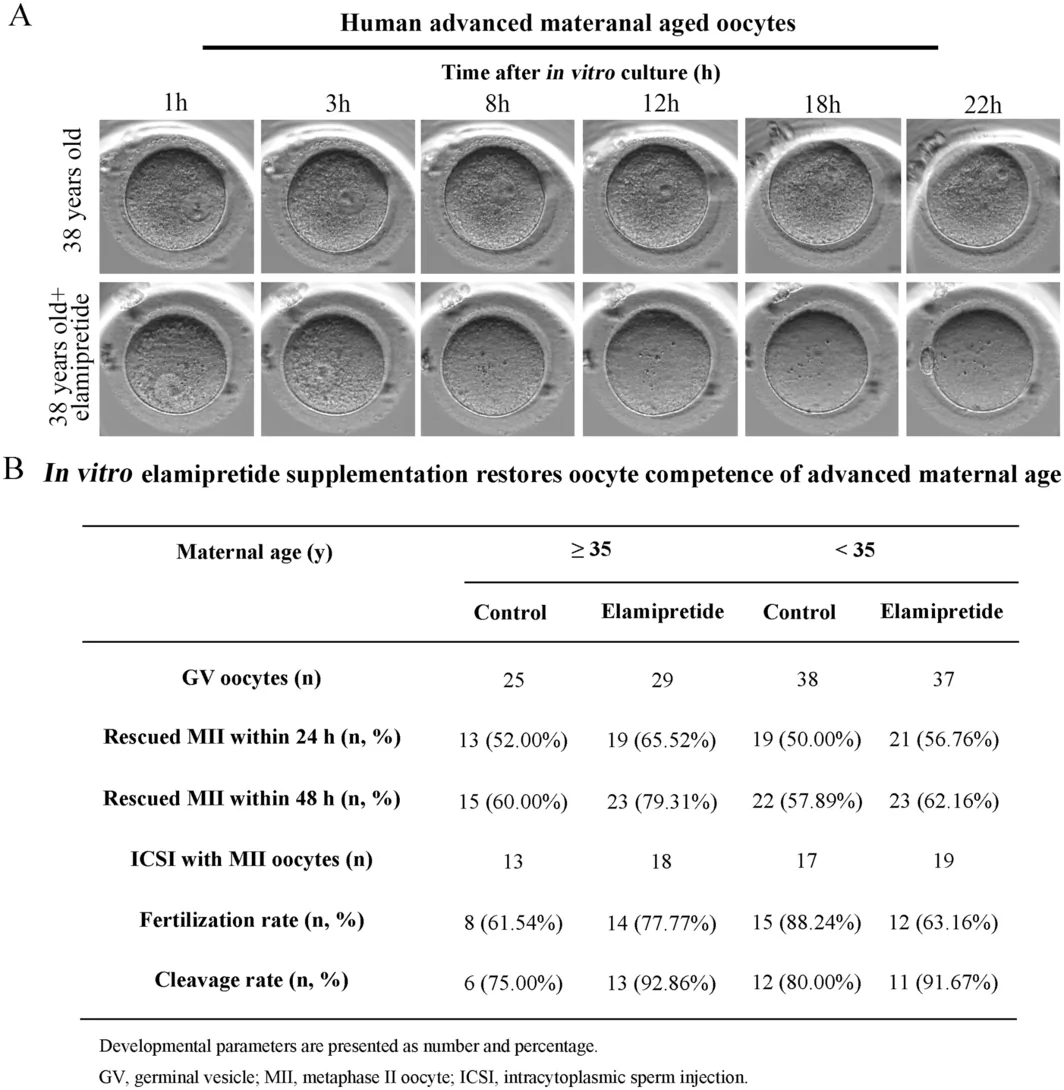
Elamipretide treatment improves human oocyte maturation, fertilization, and cleavage. (A) Elamipretide supplementation enhances oocyte maturation and first polar body extrusion in advanced maternal age women shown with time lapse microscopy. Scale bar, 20 μm. (B) In vitro elamipretide supplementation restores MII oocytes within 24 h, 48 h, fertilization rate, and cleavage rate from women, especially with advanced maternal age (more than 35 years old).

### 3.9 Elamipretide Treatment Improves Porcine Oocyte Maturation Under Oxidative Stress

We then employed porcine oocytes to determine whether elamipretide promoted maturation across species under oxidative stress by H_2_O_2_ treatment. As shown in Figure 9A, H_2_O_2_ exposure markedly reduced PBI extrusion in porcine oocytes, whereas elamipretide supplementation restored the maturation rate of porcine oocytes. 1 mM emerged as the optimal treatment concentration (Figure 9B). We found that elamipretide reduced the oxidative‐stress level of H_2_O_2_‐treated porcine oocytes (Figure 9C), and the statistical analysis aligned with the staining results (Figure 9D). Given the link between oxidative stress and mitochondrial function, we examined mitochondrial distribution in porcine oocytes and found that elamipretide reversed the H_2_O_2_‐induced loss of cortical mitochondria (Figure 9E). Moreover, LC3 expression was restored after elamipretide treatment in H_2_O_2_‐induced porcine oocytes (Figure 9F). Elamipretide also repaired the abnormal high apoptosis induced by H_2_O_2_ treatment (Figure 9G). These results demonstrated that the ability of elamipretide to alleviate oxidative stress and restore oocyte developmental quality is functionally conserved in pigs.

**FIGURE 9:**
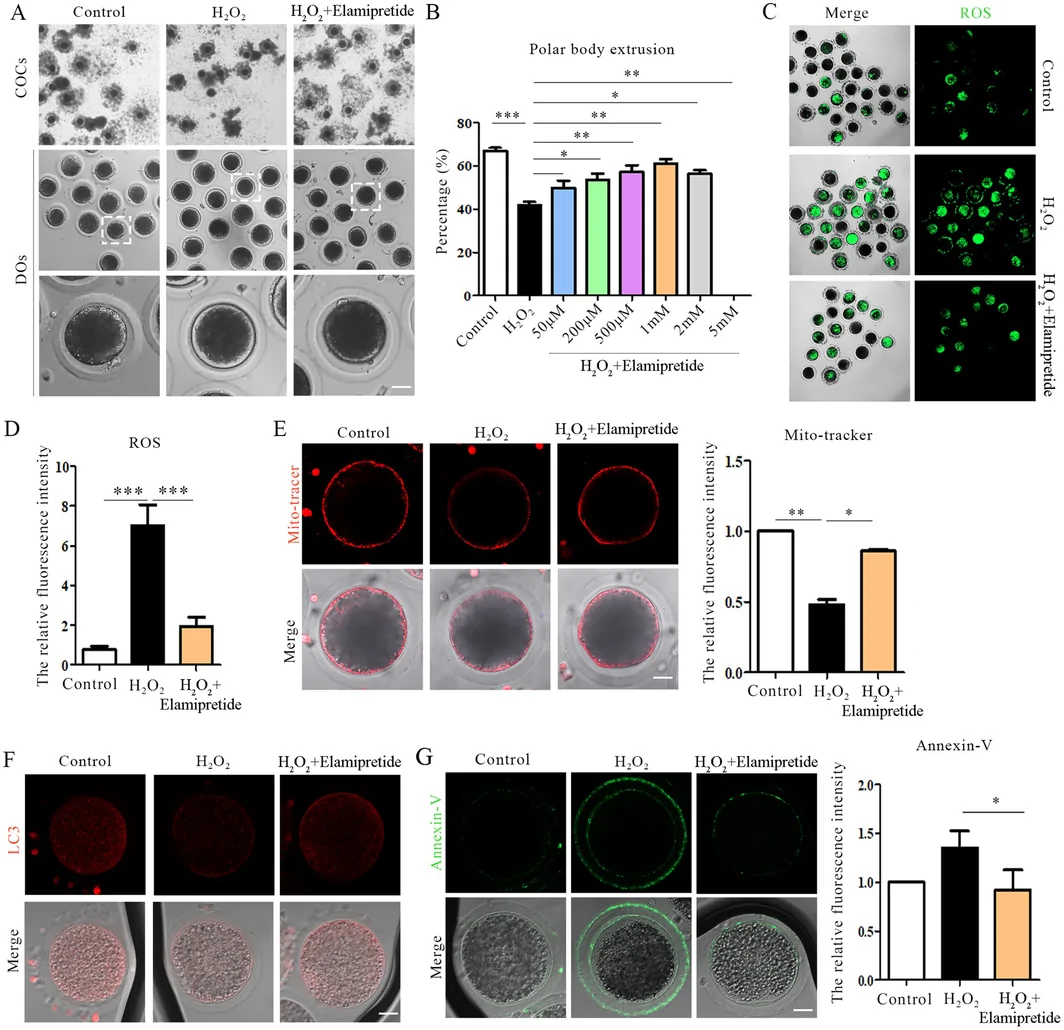
Elamipretide improves oocyte quality under oxidative stress in pigs. (A) Elamipretide improved the maturation quality of porcine oocytes treated with hydrogen peroxide for oxidative stress. (B) A concentration‐gradient assay identified 1 mM as the optimal treatment concentration for the polar body extrusion of porcine oocytes under oxidative stress. (C) The abnormally elevated ROS induced by hydrogen peroxide treatment was significantly alleviated after elamipretide treatment. Green, ROS; scale bar, 20 μm. (D) The statistical analysis of ROS level in porcine oocytes under oxidative stress treated with elamipretide. (E) Elamipretide reversed the mitochondrial fluorescence intensity reduced by hydrogen peroxide treatment to normal levels in porcine oocytes. Red, mitochondria; scale bar, 20 μm. (F) Elamipretide reversed hydrogen‐peroxide‐induced autophagy suppression in porcine oocytes. Red, LC3; scale bar, 20 μm. (G) Elamipretide mitigated the apoptosis surge triggered by oxidative stress in porcine oocytes. Green, Annexin‐V; scale bar, 20 μm. *p < 0.05, **p < 0.01, ***p < 0.001.

## 4 Discussion

In present study, we proposed a novel peptide therapy method with elamipretide to reverse ovary aging for female fertility: we showed that elamipretide promoted nuclear and cytoplasmic maturation of aged oocytes, and elamipretide reversed age‐related decline in oocyte quality through Vitamin B6‐VEGF axis and ultimately improves reproductive outcomes (Figure 10).

**FIGURE 10:**
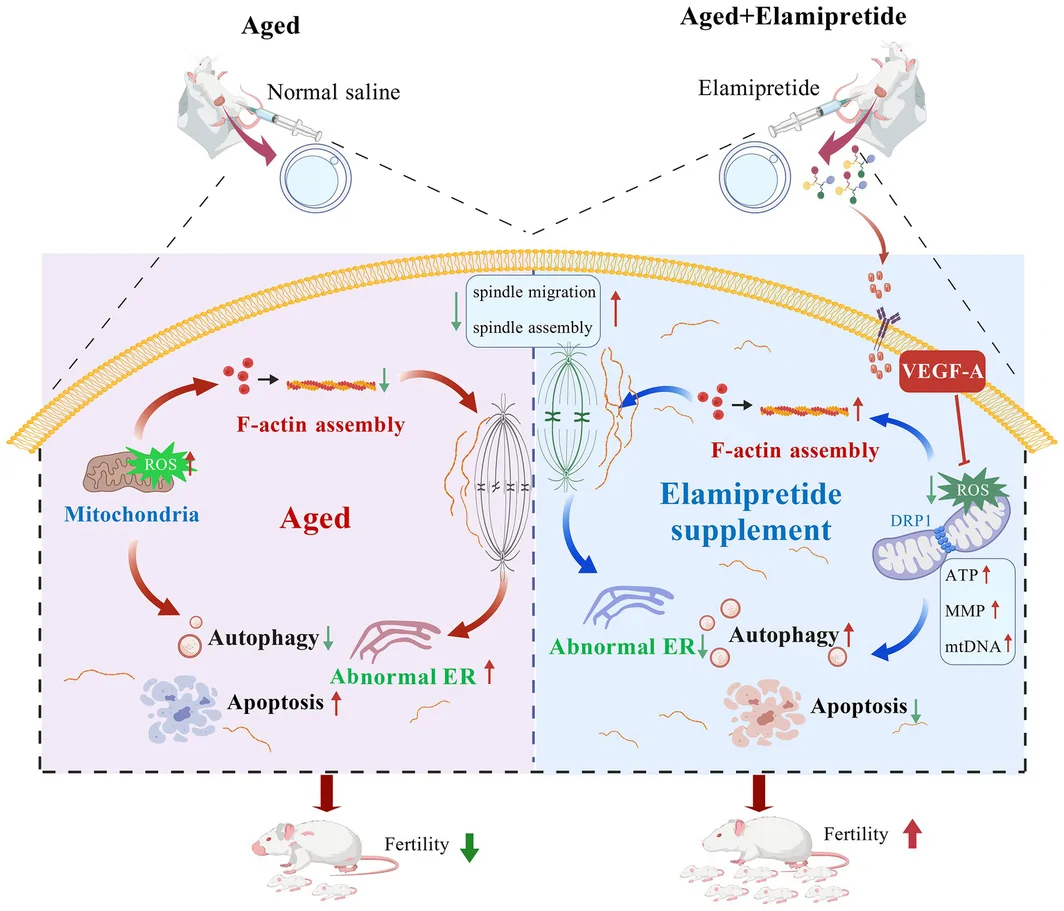
Diagram for the effects of elamipretide supplement on female fertility of aged mice. Elamipretide promoted nuclear and cytoplasmic maturation of aged oocytes, showing with restored cytoskeleton integrity, mitochondrial function, and organelle homeostasis; and elamipretide reversed age‐related decline in oocyte quality through Vitamin B6‐VEGF axis and ultimately improves reproductive outcomes (Created with BioGDP.com).

We first verified the therapeutic effect of elamipretide and explored the optimal treatment regimen. Previous studies have demonstrated that the injection of elamipretide into mice at concentrations of 3 and 5 mg/kg has the best effect (Gu et al. 2025; Kingren et al. 2024; Zhang et al. 2017). We found that the 5 mg/kg concentration of elamipretide had the best therapeutic effect on reproduction, and continuous injection of elamipretide for 7 days could effectively improve the fertility of mice aged 8, 10 months, which was confirmed by ovarian follicles, oocyte fertilization and embryonic development ability. Moreover, human oocyte in vitro culture data also confirmed that elamipretide significantly improved the human GV oocyte maturation, fertilization, and early embryo quality. To confirm the effects of elamipretide on oocyte quality, we evaluated the effects of elamipretide on actin filaments and microtubules in aged oocytes, which are the core drivers for the nuclear maturation of oocytes. The post‐ovulatory aging porcine oocytes exhibited abnormal levels of cortical F‐actin and acetylation of α‐tubulin, which subsequently led to spindle assembly defects (Bai et al. 2024; Park et al. 2025). We found that the microtubule stability and spindle polarity formation were both disrupted, which led to abnormal spindle morphology and chromosomal arrangement in oocytes of aged mice, and elamipretide supplementation could rescue the impairments. Other anti‐aging agents such as MitoQ or BGP‐15 also reversed the spindle and chromosome abnormalities observed in reproductively aged female mice (Al‐Zubaidi et al. 2021). We also found that elamipretide could restore cytoplasmic actin in aged oocytes, which is consistent with another study showing that treatment with elamipretide promotes the rapid repair of ATP‐dependent processes and facilitates the recovery of actin cytoskeleton and cell polarity (Birk et al. 2013). Besides, it is shown that elamipretide treatment reduced markers of parietal epithelial cell activation including Collagen IV, pERK1/2, and α‐smooth muscle actin, and improved cytoskeletal integrity (Sweetwyne et al. 2017).

Mitochondrial dysfunction in aged oocytes resulted in insufficient energy supply to support spindle dynamics, thereby including spindle assembly defects (Mihalas et al. 2024). Elamipretide is a synthetic tetrapeptide that specifically targets the inner mitochondrial membrane, with its core mechanism involving specific binding to cardiolipin (Sabbah et al. 2025), a signature phospholipid which plays a critical role in maintaining respiratory chain supercomplex assembly, membrane stability, and energy transduction (Zhang et al. 2025). Elamipretide binds to cardiolipin through electrostatic and hydrophobic interactions, reducing the negative surface charge density of the membrane and thereby modulating its physical properties (Mitchell et al. 2022). This binding inhibits the peroxidase activity of cytochrome c, mitigates cardiolipin oxidative damage, and preserves the integrity of the mitochondrial membrane (Mitchell et al. 2020). Our transcriptome analysis also revealed that genes enriched for inner mitochondrial membrane organization, mitochondrial metabolism, and oxidative‐stress response were significantly changed after elamipretide treatment in aged oocytes. We showed that the addition of elamipretide could restore the expression of mitochondrial related genes and mitochondrial localization, increase the mitochondrial copy number in oocytes, mediate the recovery of intracellular ATP levels and MMP, and reduce intracellular ROS levels, indicating that elamipretide can improve mitochondrial function of aged oocytes. Besides, the supplement of elamipretide in porcine oocytes under oxidative stress induced by H_2_O_2_ also confirmed its roles on mitochondria function and ROS control with pig model. In a headache male mouse model, elamipretide could maintain intracellular mitochondrial homeostasis by regulating MMP through the Sirt3/Pgc‐1α pathway (Shan et al. 2023). Elamipretide is also able to target cytochrome c in the mitochondrial inner membrane of damaged mitochondrial dysfunction of cardiomyocytes (CMs), and inhibits pathological ROS production (Zheng et al. 2022). During porcine in vitro embryo production, elamipretide supplementation effectively mitigated oxidative stress damage, augmented oocyte antioxidant capacity, and elevated the maturation rate and blastocyst formation rate (Nguyen et al. 2025). In aged human bone marrow‐derived mesenchymal stem cells (BM‐MSCs), elamipretide intervention effectively restored the functional homeostasis of mitochondria, markedly reduced intracellular ROS levels, enhanced oxygen consumption rate, and thereby reversed age‐related functional decline of BM‐MSCs (Duan et al. 2025). Elamipretide encompasses the mitigation of oxidative stress, the suppression of inflammatory processes, and maintaining mitochondrial dynamics (Du et al. 2024). Treatment with elamipretide improved mitochondrial respiratory capacity and promoted supercomplex organization, which benefits on improving cardiac mitochondrial dysfunction in the model of Barth Syndrome mice (Russo et al. 2022). Therefore, the mitochondria‐targeted peptide elamipretide can significantly improve the maturation quality of aged oocytes by targeting and regulating mitochondrial function.

Normal organelle function is a fundamental prerequisite for the cytoplasmic maturation of oocytes. We found that elamipretide can restore Golgi apparatus functions, and elamipretide induced a reverse in ER stress level mediated by GRP78, restored the normal distribution of lysosomes, and reduced the level of autophagy in aged oocytes. The treatment with elamipretide can inhibit the release of cytokines in skeletal muscle cells mediated by the Golgi apparatus (Lightfoot et al. 2015), and treatment with elamipretide can reduce ER stress in leukocytes from Type 2 diabetes in patients (Escribano‐Lopez et al. 2019). Elamipretide mediates the regulation of autophagy levels by regulating cPLA2‐induced lysosomal membrane permeability in rat (Zhang, Chen, et al. 2023). Elamipretide also promotes the formation of autolysosomes and autophagosomes, thereby facilitating autophagic flux to a certain degree in human hepatocellular carcinomas (Mo et al. 2024). Besides, elamipretide was reported to promote PHB2‐mediated mitophagy activation to inhibit mtDNA release in mouse microglial cell (BV2) (Ji et al. 2024). Thus, elamipretide can effectively ameliorate oocyte quality by repairing organelle dysfunction in aged oocytes.

Subsequently, we tried to explore the molecular mechanisms underlying the improvement of female fertility by elamipretide. Metabolomics data indicated that elamipretide treatment could disturb the overall metabolite levels in the ovary of aged female mice. Aging leads to an overall decrease in the levels of granulosa cell metabolites, especially the pyruvate levels (Shi et al. 2025). We showed that the level of pyruvate significantly changed, revealing that pyruvate may be a key substance for the decline in fertility in advanced females. Pyruvate is an important component of the TCA cycle and is directly related to energy metabolism, which also suggests that changes in mitochondrial‐mediated energy metabolism levels may be an important cause of infertility during female aging, which are also noted by another study (Barzilai et al. 2012; Hipkiss 2008). Our KEGG analysis also revealed that the metabolism of Vitamin B6 was significantly altered after elamipretide treatment in aged mice, suggesting that Vitamin B6 may play an important role in improving the fertility of aged mice with elamipretide. Pyridoxal 5′‐phosphate (PLP) is the active form of vitamin B6 (Stach et al. 2021). PLP deficiency in HEK293 cells caused accumulation of lactate and pyruvate along with decreased tricarboxylic acid (TCA) cycle intermediates, thereby impairing mitochondrial oxidative metabolism (Ciapaite et al. 2023). Vitamin B6 (pyridoxine) was demonstrated to synergize with vitamin B3 (nicotinamide) in regulating key molecular pathways of skeletal muscle maintenance and regeneration, thereby ameliorating age‐related sarcopenia (Nowacka et al. 2025). Pyruvate has been proposed as an anti‐aging metabolite that improves skin aging by generating NAD in dermal fibroblasts, thereby regulating mitochondrial and lysosome functions (Kim et al. 2018). We showed that direct supplementation of Vitamin B6 can improve the oocyte quality of aged mice.

Besides, our transcriptome analysis also found that the gene changes related to the TCA cycle, which were consistent with our metabolome results. We also screened out potential molecular pathways and identified the vascular endothelial growth factor (VEGF) pathway as a key target. In aortic stenosis treatment, VEGF protects cardiac function in myocardial infarction models via the canonical VEGF‐PI3K‐AKT signaling pathway as well as the mitochondrial anti‐apoptotic pathway (Xu et al. 2011). Elamipretide has been demonstrated to conjugate with anti‐VEGFR2 and exert therapeutic effects in diabetic nephropathy models by reducing pro‐inflammatory factors and fibrosis markers, as well as alleviating oxidative stress (Liu et al. 2023). Vitamin B6 was found to promote AMP‐activated protein kinase (AMPK) phosphorylation and VEGF‐A synthesis in human umbilical vein endothelial cells (Wang et al. 2025). We showed that elamipretide restored the expression of VEGF‐A and its downstream PI3K/AKT proteins. Treatment with PTC299 to inhibit VEGF‐A transcriptional activity on aged oocytes showed similar effects to elamipretide and improved the quality of aging oocytes. However, treatment with the PI3K inhibitor LY294002 and wortmannin showed no significant rescue effects on aged oocyte maturation, further indicating that VEGF is the key target for elamipretide. Therefore, we speculate that elamipretide regulates the metabolic process of vitamin B6 via the VEGF pathway.

In summary, our study showed that short‐term treatment with elamipretide can significantly improve the fertility of aged female mice, and it is mainly through effects on the rescue of cytoplasmic and nuclear maturation of oocytes by vitamin B6‐based VEGF pathway.

## Author Contributions

H.‐L.Z., S.‐C.S. designed the study. H.‐L.Z., Y.W., C.W., X.G. performed the experiments. H.C., Y.‐X.H., X.W., Z.‐J.W., W.‐L.P. R.‐J.M., P.‐S.L., J.S. contributed the materials. H.‐L.Z., Y.W., S.‐C.S. wrote the manuscript. H.‐L.Z., Y.W., C.W., S.‐C.S. analyzed the data. All the authors approved the final manuscript.

## Funding

This work was supported by the National Key Research and Development Program of China (2023YFD1300502), the Fundamental Research Funds for the Central Universities of China (KJJQ2026001, RENCAI2025035); Open Competition Project of Guangxi Plateau Discipline of Youjiang Medical University for Nationalities (YY2026GYZ15, YY2026GYZ08).

## Ethics Statement

All use procedures of animals and experimental programs were carried out in accordance with the guidelines of the Animal Research Ethics Committee of Nanjing Agricultural University and approved by the Animal Research Ethics Committee. Human oocyte collection was followed to the guideline of Ethic Committee of Maternity and Child Health Care of Guangxi Zhuang Autonomous Region, and the patients were aware and permitted the use of the oocytes.

## Consent

All authors consent for the publication of this study.

## Conflicts of Interest

The authors declare no conflicts of interest.

## Supporting information


**Figure S1:** Elamipretide orchestrates cortical‐granule trafficking in aged mouse oocytes. (A) Comparison of body and ovarian weights between aged and young mice. (B) The restorative effect of elamipretide on litter size is transient. (C) Elamipretide reversed aging‐induced defects in cortical granule trafficking. Green, cortical granule; blue, DNA; scale bar, 20 μm. ***p < 0.001, ****p < 0.0001.
**Figure S2:** Elamipretide fails to modulate aged oocyte quality through the PI3K/AKT pathway. (A) PI3K/AKT inhibitor LY294002 only partially restored aged oocytes maturation. (B) LY294002 treatment recovered the abnormally elevated cytoplasmic actin levels in aged oocytes. Gray, cytoplasmic actin; red, cortical actin; scale bar, 20 μm. (C) LY294002 failed to mitigate the heightened apoptosis observed in aged oocytes. (D) Another PI3K/AKT inhibitor Wortmannin failed to reverse ROS levels in aged oocytes. Green, ROS; scale bar, 20 μm. (E) The statistical analysis of PTC299 reversing ROS levels in aged oocytes. (F) The VEGF inhibitor PTC299 reversed the decline in cytoplasmic actin levels in age oocytes. Red, actin; blue, DNA; scale bar, 20 μm. *p < 0.05, **p < 0.01, ****p < 0.0001, and ns, no significant difference.
**Table S1:** Primers used in the study.

## Data Availability

All data generated or analyzed during this study are included in this published article.
