# Supplementary material for: Elamipretide Rejuvenates Oocyte Quality and Restores Female Fertility During Reproductive Aging
Source: Aging Cell. 2026 Oct 2;25(10):e70749. doi: 10.1111/acel.70749 (PMC13633435; doi:10.1111/acel.70749)
Supplement: Supplementary file 1 [file ACEL-25-e70749-s001.docx]

**Supplemental Materials for**

**Elamipretide rejuvenates oocyte quality and restores female fertility during reproductive aging**

Hao-Lin Zhang^1#^, Yue Wang^1#^, Caizhu Wang^2#^, Xin Guo^1^, Huanhua Chen^2^, Yu-Xuan Hou^1^, Xuan Wu^1^, Zi-Jian Wu^1^, Wen-Lin Pan^1^, Rui-Jie Ma^1^, Ping-Shuang Lu^1^, Jinhui Shu^2^, Shao-Chen Sun^1, 3*^

^1^College of Animal Science and Technology, Nanjing Agricultural University, Nanjing 210095, China.

^2^Center of Reproductive Medicine, Maternity and Child Health Care of Guangxi Zhuang Autonomous Region, Nanning, Guangxi 530003, China.

^3^Guangxi Key Laboratory of Artificial Intelligence for Genetic Diseases of Long-dwelling Nationalities, Affiliated Hospital of Youjiang Medical University for Nationalities, Baise, Guangxi 533000, China.

**Running Title:** Elamipretide for reproductive aging

^#^These authors contributed equally to this work.

**^*^Correspondence to:** Shao-Chen Sun, College of Animal Science and Technology, Nanjing Agricultural University, Nanjing, China. E-mail: [sunsc@njau.edu.cn](mailto:sunsc@njau.edu.cn)

**Figure S1.** Elamipretide orchestrates cortical-granule trafficking in aged mouse oocytes. (A) Comparison of body and ovarian weights between aged and young mice. (B) The restorative effect of elamipretide on litter size is transient. (C) Elamipretide reversed aging-induced defects in cortical granule trafficking. Green, cortical granule; blue, DNA; scale bar, 20μm. *** P < 0.001, **** P < 0.0001.

**Figure S2.** Elamipretide fails to modulate aged oocyte quality through the PI3K/AKT pathway. (A) PI3K/AKT inhibitor LY294002 only partially restored aged oocytes maturation. (B) LY294002 treatment recovered the abnormally elevated cytoplasmic actin levels in aged oocytes. Grey, cytoplasmic actin; red, cortical actin; scale bar, 20μm. (C) LY294002 failed to mitigate the heightened apoptosis observed in aged oocytes. (D) Another PI3K/AKT inhibitor Wortmannin failed to reverse ROS levels in aged oocytes. Green, ROS; scale bar, 20μm. (E) The statistical analysis of PTC299 reversing ROS levels in aged oocytes. (F) The VEGF inhibitor PTC299 reversed the decline in cytoplasmic actin levels in age oocytes. Red, actin; blue, DNA; scale bar, 20μm. * P < 0.05, ** P < 0.01, **** P < 0.0001, and ns, no significant difference.

**Table S1. Primers used in the study.**

| Drp1 | F 5'-CAGGTGGTGGGATTGGAGAC | R 5'-CTGGCATAATTGGAATTGGTTT |  |
| --- | --- | --- | --- |
| Opa1 | F 5'-CCGAGGATAGCTTGAGGGTT | R 5'-CGTTCTTGGTTTCGTTGTGA |  |
| PGC1-α | F 5'-TATGGAGTGACATAGAGTGTGCT | R 5'-CCACTTCAATCCACCCAGAAAG |  |
| ATP5B | F 5'-GGTTCATCCTGCCAGAGACTA | R 5'-AATCCCTCATCGAACTGGACG |  |
| SOD2 | F 5'-CAGACCTGCCTTACGACTATGG | R 5'-CTCGGTGGCGTTGAGATTGTT |  |
| Gpx3 | F 5'-CCTTTTAAGCAGTATGCAGGCA | R 5'-CAAGCCAAATGGCCCAAGTT |  |
| Ndufs3 | F 5'-TGGCAGCACGTAAGAAGGG | R 5'-CTTGGGTAAGATTTCAGCCACAT |  |
| Caspase3 | F 5'-ATGGAGAACAACAAAACCTCAGT | R 5'-TTGCTCCCATGTATGGTCTTTAC |  |
| Jun | F 5'-CCTTCTACGACGATGCCCTC | R 5'-GGTTCAAGGTCATGCTCTGTTT |  |
| BAX | F 5'-TGAAGACAGGGGCCTTTTTG | R 5'-AATTCGCCGGAGACACTCG |  |
| PARP1 | F 5'-GGCAGCCTGATGTTGAGGT | R 5'-GCGTACTCCGCTAAAAAGTCAC |  |
| Capn1 | F 5'-ATGACAGAGGAGTTAATCACCCC | R 5'-GGCTATGAGAAACCGGAGGG |  |
| GM130 | F 5'-TGTCACAGAACCGAGAGCTGA | R 5'-GCTGCTCCTGTAGACCCTG |  |
| Rab11 | F 5'- CTCTGGACGAGGTCTTCCG | R 5'-TGTTCCGTGTGAACTGGATGG |  |
| GRP78 | F 5'-ACTTGGGGACCACCTATTCCT | R 5'-ATCGCCAATCAGACGCTCC |  |
| ERO1L | F 5'-TTCTGCCAGGTTAGTGGTTACC | R 5'-GTTTGACGGCACAGTCTCTTC |  |
| CHOP | F 5'-CTGGAAGCCTGGTATGAGGAT | R 5'-CAGGGTCAAGAGTAGTGAAGGT |  |
| ATF4 | F 5'-ATGGCGCTCTTCACGAAATC | R 5'-ACTGGTCGAAGGGGTCATCAA |  |
| LC3 | F 5'-GACCGCTGTAAGGAGGTGC | R 5'-CTTGACCAACTCGCTCATGTTA |  |
| ATG7 | F 5'-GTTCGCCCCCTTTAATAGTGC | R 5'-TGAACTCCAACGTCAAGCGG |  |
| NRBF2 | F 5'-AAGGACCCCTCAACCTTGCT | R 5'-CAGTTCCAGTGATAAGTGAGCC |  |
| LAMP2 | F 5'-TGTATTTGGCTAATGGCTCAGC | R 5'-TATGGGCACAAGGAAGTTGTC |  |
| Prkcb | F 5'-AAGGAGCATGCGTTTTTCCG | R 5’-GTCTCGCTTGTCTCTAGCTTTTG | |
| Akt2 | F 5’-CGCCGGTGACAGACGATA | R 5’-TTTGTGGAGCCAGCCTTCTT |  |
| Pxn | F 5’-GACGACCTCGATGCCCTG | R 5’-GGGGCTCCTCTGACAAGAAC |  |
| Rac3 | F 5’-TATCCCCACAGTTTTCGACAAC | R 5’-GAGAGTGGCCGAAGCCTAT | |
| Pik3cd | F 5’-TTAATCTCCCAGGCAGAGGG | R 5’-CTGAGGTCTGTTTTCCTGTTTGG |  |
